# Generation and characterization of HLA-universal platelets derived from induced pluripotent stem cells

**DOI:** 10.1038/s41598-020-65577-x

**Published:** 2020-05-21

**Authors:** Phatchara Norbnop, Praewphan Ingrungruanglert, Nipan Israsena, Kanya Suphapeetiporn, Vorasuk Shotelersuk

**Affiliations:** 1 Doctor of Philosophy Program in Medical Sciences, Faculty of Medicine, Chulalongkorn University, Bangkok, 10330 Thailand; 2 Center of Excellence for Medical Genomics, Department of Pediatrics, Faculty of Medicine, Chulalongkorn University, Bangkok, 10330 Thailand; 3 Stem Cell and Cell Therapy Research Unit, Faculty of Medicine, Chulalongkorn University, Bangkok, 10330 Thailand; 4 Department of Pharmacology, Faculty of Medicine, Chulalongkorn University, Bangkok, 10330 Thailand; 5 Excellence Center for Genomics and Precision Medicine, King Chulalongkorn Memorial Hospital, the Thai Red Cross Society, Bangkok, 10330 Thailand

**Keywords:** Pluripotent stem cells, Coagulation system

## Abstract

Platelet demand has increased around the world. However, the inadequacy of donors, the risk of transfusion-transmitted infections and associated reactions, and the refractory nature of platelet transfusions are among the limitations of allogeneic platelet transfusions. To alleviate these problems, we propose generating platelets in a laboratory that do not induce alloimmunity to human leukocyte antigen (HLA) class I, which is a major cause of immune reaction in platelet transfusion refractoriness. Induced pluripotent stem cells (iPSCs) were generated from peripheral blood mononuclear cells (PBMCs) of a healthy Thai woman. We then knocked out the β2-microglobulin (*β2m*) gene in the cells using paired CRISPR/Cas9 nickases and sequentially differentiated the cells into haematopoietic stem cells (HSCs), megakaryocytes (MKs) and platelets. Silencing of HLA class I expression was observed on the cell surface of *β2m*-knockout iPSCs, iPSC-derived HSCs, MKs and platelets. The HLA-universal iPSC-derived platelets were shown to be activated, and they aggregated after stimulation. In addition, our *in vivo* platelet survival experiments demonstrated that human platelets were detectable at 2 and 24 hours after injecting the *β2m-KO* MKs. In summary, we successfully generated functional iPSC-derived platelets *in vitro* without HLA class I expression by knocking out the *β2m* gene using paired CRISPR/Cas9 nickases.

## Introduction

Platelets are nonnucleated cells that play important roles in thrombosis and haemostasis^1^. Patients with thrombocytopenia or platelet dysfunction usually require a platelet transfusion from donors to prevent the risk of life-threatening haemorrhage^2^. Because of the rise of ageing populations, the incidence of haematological malignancy, and haematopoietic stem cell transplantation, among many other factors, platelet demand has increased in recent decades around the world^3^. In addition to the inadequacy of donors and the costly process of preparing donated platelets^4,5^, transfusion-transmitted infection and the subsequent reaction pose limitations regarding donor platelet transfusion^6^. Moreover, recipients may experience inadequate responses to platelet transfusion (platelet transfusion refractoriness) caused by alloimmunity against HLA class I^7–9^. Although finding HLA-matched donors or cross-matched platelets could decrease these problems^4,10^, HLA-matched platelet transfusion is still unsuccessful in 20–50% of severely alloimmunized patients^11^. The generation of universal platelets would help reduce the incidence of these unfavourable outcomes.

β2-microglobulin (β2M) is a light chain member of the HLA class I molecules, and it is necessary for HLA class I assembly and presentation on the cell membrane. Elimination of the β2-microglobulin (*β2m*) gene leads to the absence of HLA class I expression on the cell surface^12,13^. Previous attempts have successfully generated HLA-universal platelets *in vitro* by disrupting the *β2m* gene with RNA interference (RNAi) technology in CD34 + progenitor cells and induced pluripotent stem cells (iPSCs)^14–16^ or with transcription activator-like effector nuclease (TALEN) technologies in iPSCs^17^. In addition to unlimited self-renewal, proliferation, differentiation, and the ability to grow in culture media, iPSCs can be easily generated from blood cells or other somatic cells with different immunologic backgrounds to avoid non-HLA class I rejection^18^. Therefore, iPSCs are considered an appropriate unlimited cell source for HLA-universal platelet generation.

Targeted genome editing technologies have rapidly advanced^19–22^. Compared to the CRISPR/Cas9 nuclease system, paired CRISPR/Cas9 nickases have been shown to increase genome editing specificity^23^, and they provide comparable or higher on-target efficiency with barely detectable off-target effects^24^. Here, we successfully generated HLA class I-deficient iPSCs by knocking out the *β2m* gene using paired CRISPR/Cas9 nickases, and then we differentiated the cells *in vitro* into functional platelets without HLA class I expression.

## Results

### Generation and characterization of HLA class I-universal iPSCs

**Figure 1:**
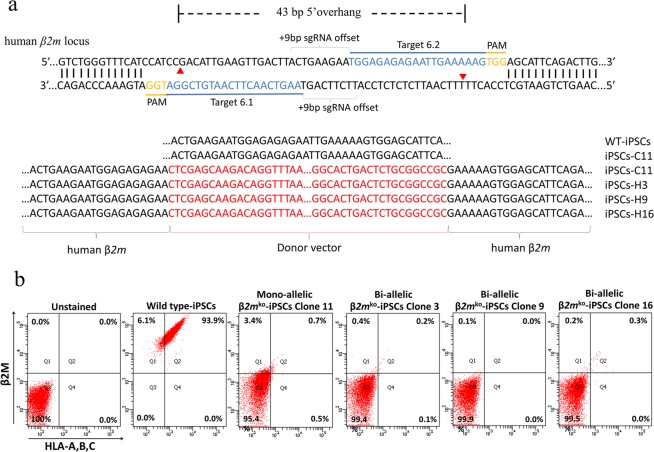
Generation and characterization of HLA class I-universal iPSCs. Schematic diagram showing the two gRNAs with a 9 bp offset designed to target exon 2 of human *β2M*; Sanger sequencing showing *β2M* sequences of the wild-type allele of WT-iPSCs and iPSCs-C11. The donor sequences in the *β2M* sequences were found in iPSCs-C11, iPSCs-H3, iPSCs-H9, and iPSCs-H16 (**a**). Flow cytometry dot plot of HLA class I and β2M expression in all lines of iPSCs (**b**).

Two wild-type human iPSC clones were isolated, and only one clone survived. This clone was used in all experiments. To knock out *β2m*, paired CRISPR/Cas9 nickases and a donor vector (Supplementary Fig. 1a) were transfected into healthy human iPSCs using nucleofection (Supplementary Fig. 1b). A pair of sgRNAs (Target 6.1 and Target 6.2) was designed with a 9 base-pair (bp) offset, as indicated in Fig. 1a. A T7E1 assay revealed the targeting efficiency of the paired CRISPR/Cas9 nickases and the expected PCR product sizes of 228 and 320 bp in the transfected group (Supplementary Fig. 2). After transfection of both paired CRISPR/Cas9 nickases and donor vectors into human wild-type iPSCs, cells were grown under hygromycin selection conditions, and many iPSC clones survived. These clones were selected for subsequent culture and analysis by PCR amplification to detect donor recombination. The wild-type iPSCs showed only the wild-type allele, as indicated by a PCR product size of 548 bp. In addition to the 548-bp band, one clone, iPSC-C11, had left and right donor recombination bands of 796 and 858 bp, respectively. Other clones, iPSCs-H3, iPSCs-H9, and iPSCs-H16, exhibited only the left and right donor recombination bands without the wild-type allele band, as shown in Supplementary Fig. 3. The PCR products were sent for Sanger sequencing. All donor recombination bands revealed a donor sequence between the *β2m* sequence, and the wild-type allele bands had only the *β2m* sequence, as indicated in Fig. 1a.

HLA and β2M cell surface expression on iPSCs was measured by flow cytometry. A total of 99.9% of the wild-type iPSCs expressed β2M, and 96.6% of them expressed HLA-A, B, and C. In contrast, 95.1%, 99.5%, 100%, and 99.6% of clones iPSCs-C11, iPSCs-H3, iPSCs-H9, and iPSCs-H16, respectively, did not express either HLA-A, B, C or β2M, as shown in Fig. 1b. All iPSC lines expressed the pluripotency markers OCT4, NANOG, SSEA-4, TRA-1-60 and TRA-1-81 (Supplementary Fig. 4a). Their pluripotency was verified by their abilities to form embryoid bodies with the three germ layers; marker expression verified the differentiation, which was α-fetoprotein for endoderm, Brachyury for mesoderm and Nestin for ectoderm (Supplementary Fig. 4b). In addition, wild-type iPSCs and all four iPSC lines maintained an embryonic stem cell-like morphology with normal karyotypes (Supplementary Fig. 4c).

### Generation and characterization of haematopoietic stem cells, megakaryocytes and platelets from HLA class I-universal iPSCs

**Figure 2:**
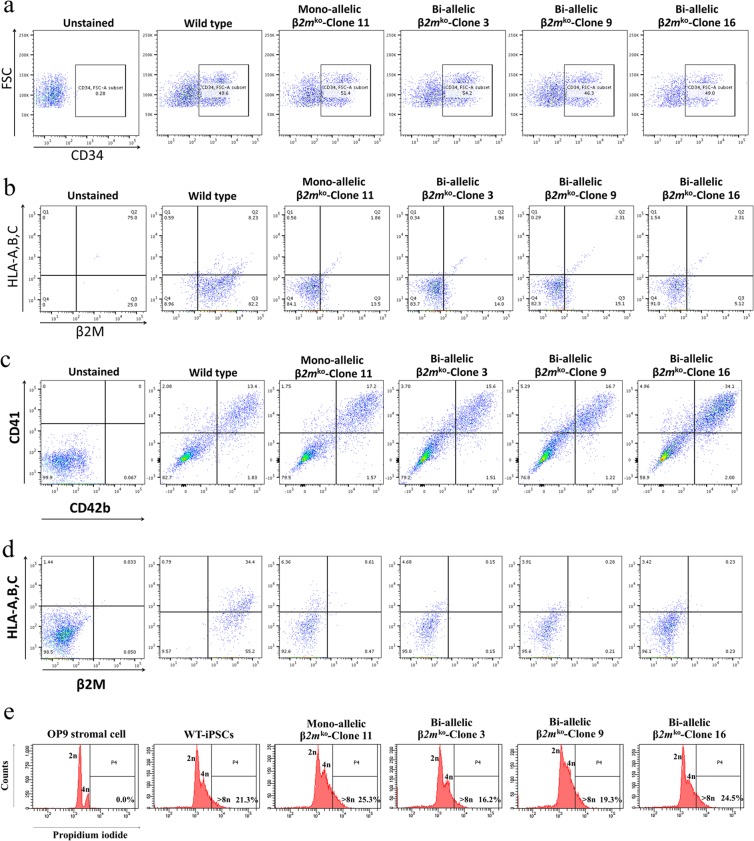
Flow cytometry dot plot of progenitor cells isolated from ES-sacs showing CD34 expression (**a**) and HLA-A, B, C and β2M expression in all iPSC-derived haematopoietic stem cell lines (**b**). Detection of HLA class I and *β2M* expression in megakaryocytes and their characterization. MKs were differentiated from wild-type iPSCs, monoallelic *β2M*^ko^-iPSCs and three lines of biallelic *β2M*^ko^-iPSCs. Flow cytometry dot pots revealed CD41 + /CD42b + iPSC-derived MKs generated from all iPSC lines (**c**) and HLA-A, B, C and β2M expression (**d**). MK characterization by polyploidy analysis in flow cytometry histogram (**e**).

The ES-sac method was used to produce CD34 + haematopoietic stem cells (HSCs) from all five iPSC lines. ES-sac-like structures obtaining HSCs were observed from all iPSC lines after 14 days of coculture with 10T1/2 feeder cells and VEGF treatment (Supplementary Fig. 5a). Flow cytometry was used to analyse HSCs obtained from ES-sacs using APC/Cy7-conjugated anti-human CD34, PerCP-conjugated anti-human HLA-A, B, and C and APC-conjugated anti-human β2-microglobulin. HSCs from the monoallelic *β2m*^*ko*^-iPSCs and the three biallelic *β2m*^*ko*^-iPSCs exhibited differentiation rates that were similar to those of the wild-type group (Fig. 2a). Moreover, more than 82.3% of the monoallelic *β2m*^*ko*^-iPSCs and all of the biallelic *β2m*^*ko*^-HSCs did not express HLA-A, B, and C or β2M, while 90.43% of the wild-type HSCs expressed β2M, and 8.23% of them expressed HLA-A, B, or C (Fig. 2b).

HSCs were isolated from ES-sacs, and 2 × 10^5^ cells were seeded into each well of a 6-well plate. These cells were then cultured in haematopoietic cell differentiation medium supplemented with TPO, SCF and heparin. After 7 days of culture (day 21), iPSC-derived MKs of all lines were analysed by flow cytometry using FITC-conjugated anti-human CD41, PE-conjugated anti-human CD42b, PerCP-conjugated anti-human HLA-A, B, and C and APC-conjugated anti-human β2-microglobulin. The results revealed that the monoallelic *β2m*^*ko*^-iPSCs and the three lines of biallelic *β2m*^*ko*^-iPSCs could generate CD41 + /CD42b + MKs at differentiation rates similar to those of the wild-type group (Fig. 2c). In addition, 92.6% of the monoallelic β*2m*^ko^-iPSC-derived MKs and 95.0%, 95.6%, and 96.1% of the biallelic β*2m*^ko^-iPSC-derived MKs (from clones 3, 9, and 16, respectively) did not express either HLA-A, B, C or β2M; however, 89.6% of the wild-type iPSC-derived MKs expressed β2M, of which 34.4% expressed HLA-A, B, or C (Fig. 2d). In addition, analysis of cell nuclei showed that all lines of iPSC-derived MKs had similar percentages of DNA content that were higher than 8n (Fig. 2e). Wright’s stain revealed that they also displayed multilobed and enlarged nuclei (Supplementary Fig. 5b). Megakaryocyte colonies with proplatelet formation were observed by phase contrast photomicrographs (Supplementary Fig. 5c) and immunofluorescence (IF) staining (Supplementary Fig. 5d). The mono- and biallelic β*2m*^ko^-iPSC-derived MKs showed proplatelet hallmark features, including a platelet tip, a shaft, swellings, and a branch point, which were similar to those of the wild-type group.

**Figure 3:**
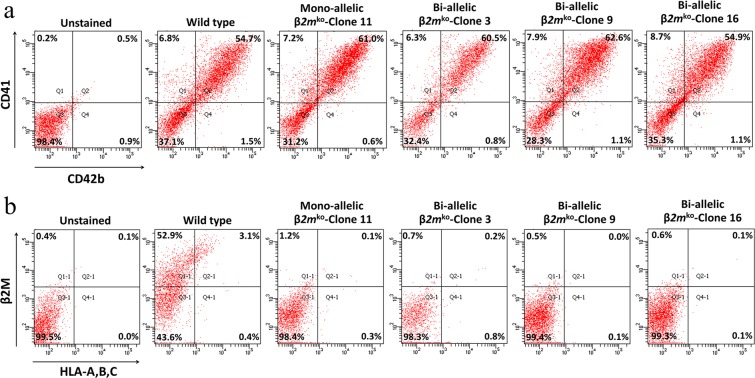
Flow cytometry showing CD41/CD42b expression (**a**) and HLA-A, B, C, and β2M expression in all lines of iPSC-derived platelets (**b**).

Platelet-specific antigens CD41 (GPIIb) and CD42b (GPIbα) were used for platelet identification. On day 24 of culture, platelet-like particles were separated from larger cells by centrifugation, and the number of platelet-like particles was determined by flow cytometry staining with FITC-conjugated anti-human CD41, PE-conjugated anti-human CD42b, PerCP-conjugated anti-human HLA-A, B, and C and APC-conjugated anti-human β2-microglobulin. The flow cytometry dot plots showed that the monoallelic *β2M*^ko^-iPSCs and all three lines of biallelic *β2M*^ko^-iPSCs could generate CD41 + /CD42b + platelets at differentiation rates that were the same as those of the wild-type group (Fig. 3a). Moreover, 98.4% of the monoallelic *β2M*^ko^-iPSC-derived platelets and 98.3%, 99.4%, 99.3% of the biallelic *β2M*^ko^-iPSC-derived platelets (from clones 3, 9, and 16, respectively) did not express HLA-A, B, C or β2M when compared with the unstained group; however, 52.9% of the wild-type iPSC-derived platelets expressed β2M, of which 3.1% expressed HLA-A, B, or C (Fig. 3b).

### Functional assessment of HLA class I-universal iPSC-derived platelets

**Figure 4:**
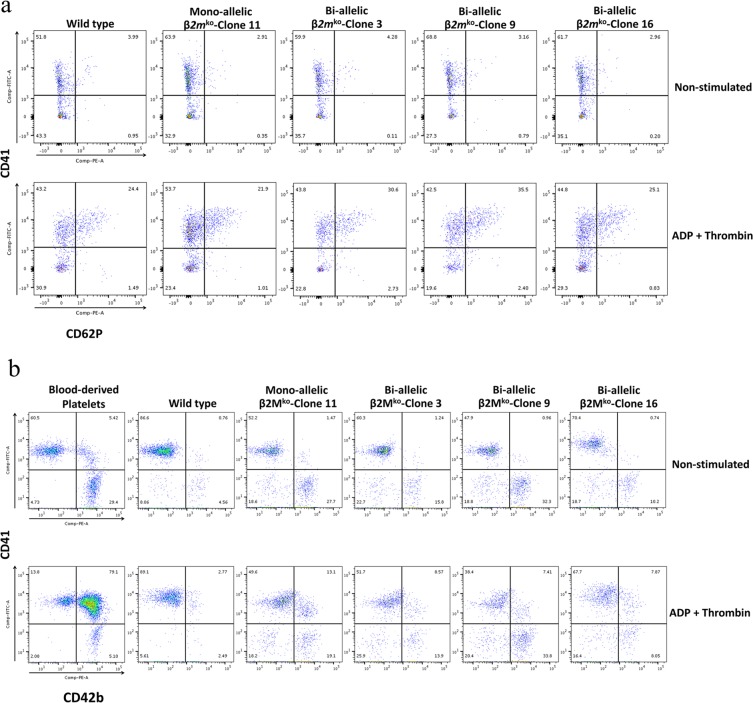
Flow cytometry analysis of platelet function measured in the presence and absence of ADP (50 µM) and thrombin (1 U/ml). Platelet activation analysis was performed by detecting CD41 and CD62P (P-selectin) expression (**a**), and platelet aggregation analysis was performed on wild-type iPSC-derived platelets and mono- and biallelic *β2M*^ko^-iPSC-derived platelets (**b**).

On day 24 of culture, all lines of iPSC-derived platelets were collected and stimulated with the classical platelet agonists ADP and thrombin. Platelet activation and aggregation were analysed using flow cytometry. The results showed that CD62P expression was increased from 2.91% of cells when non-stimulated to 21.9% in CD41 + mono-allelic *β2m*^*ko*^-iPSC-derived platelets, and the percentage of cells expressing CD62P shifted from 4.28% to 30.6%, 3.16% to 35.5%, and 2.96% to 25.1% CD41+ biallelic *β2m*^*ko*^-iPSC-derived platelet clones 3, 9 and 16, respectively. The expression frequencies were comparable with those of the wild-type group, where CD62P expression was increased from 3.99% to 24.4% when stimulated (Fig. 4a).

Platelet aggregation analysis by flow cytometry also demonstrated that the mono- and biallelic β*2m*^ko^-iPSC-derived platelets were functionally not different from wild-type iPSC-derived platelets. Flow cytometry dot plots showed that iPSC-derived platelets could aggregate with human blood-derived platelets, which were measured as dual-coloured events in quadrant 2; such aggregation events increased from 0.76% to 2.77% after stimulation by ADP and thrombin in the wild-type iPSC-derived platelet group. Similarly, an increase from 1.47% to 13.1% was observed in the monoallelic β*2m*^ko^-iPSC-derived platelets and from 1.24% to 8.57%, 0.96% to 7.41%, and 0.74% to 7.87% in the biallelic β*2m*^ko^-iPSC-derived platelets (clones 3, 9, and 16, respectively) (Fig. 4b).

**Figure 5:**
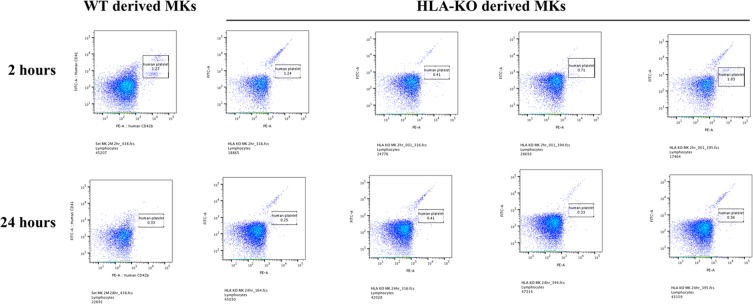
*In vivo* platelet survival assay. The percentage of human platelets in mice two and 24 hours after the administration of the *β2m*^*ko*^ MKs was 1.048 ± 0.62% and 0.34 ± 0.07%, respectively, while that of the wild-type MKs was 1.425 ± 0.54% and 0.65 ± 0.17%, respectively.

The percentage of human platelets in the mice two and 24 hours after the administration of the *β2m*^*ko*^ MKs was 1.048 ± 0.62% and 0.34 ± 0.07%, respectively, while that of the wild-type MKs was 1.425 ± 0.54% and 0.65 ± 0.17%, respectively (Fig. 5).

## Discussion

In this report, we describe a *β2M*-targeted CRISPR/Cas9 nickase approach for generating HLA class I null pluripotent stem cells. Similar to previous studies, iPSCs with *β2m* knocked out exhibit no defects in their pluripotency or differentiation potential. All our *β2m* (−/−) iPSC lines could produce functional platelets devoid of HLA class I, and they displayed *in vivo* kinetics similar to those of platelets generated from the parent lines. Our data support the *β2m* engineering strategy for generating low immunogenic cells from iPSCs. Importantly, a recent study demonstrated that double-strand breaks introduced by regular CRISPR/Cas9 techniques resulted in more mutations than what was previously estimated^24,25^. Large deletions could be detected several kilobases from the target site, which could cause unwanted consequences. The Cas9 nickase system we have employed has sufficiently high efficiency for iPSC genome engineering and should theoretically produce fewer off-target and genome toxicity effects^24,26^. Thus, unlike other genome engineering techniques that depend on double strand breaks, such as an adeno-associated virus (AAV) approach^27^, our technique does not; therefore, the approach described here may be preferable for generating engineered iPSCs intended for clinical applications until these technical issues are solved. Nevertheless, further experiments are required to validate this assumption.

The approach of generating universal cells for transplantation by knocking down MHC class I has raised the concern that cells without MHC class I may be vulnerable to natural killer (NK) cell-mediated cell lysis through missing self-responses. A previous study showed that haematopoietic donor cells with a biallelic knockout of *β2m* were eliminated by NK cells in mice^28^. For human iPSCs, it was shown that even missing one of the two groups of HLA-C (C1/C1) could trigger NK (C1/C2)-mediated cell lysis^29^. To overcome this issue, overexpressing HLA-E^27^ or HLA-C^29^ or using short hairpin RNA to reduce but not completely eliminate *β2m* levels have been suggested^30^. Nevertheless, it is still unclear whether platelets missing HLA class I are targeted by NK cells. Several lines of evidence have demonstrated that platelets can reduce the cytotoxicity of NK cells through multiple mechanisms^31,32^. Platelets with no HLA I expression were previously reported to be able to escape destruction by antibody-mediated cytotoxicity *in vitro* and *in vivo*^14–16^. Preliminary work from Koji Eto’s group suggested that MKs generated from *β2m*^*ko*^ cells, but not platelets, could activate NK cells. Our *in vivo* experiments demonstrated that human platelets were detectable at 2 and 24 hours after the injections of *β2m*^*ko*^ MKs, although they persisted at lower percentages than those of the wild-type iPSC-derived MKs. This encourages further exploration into the possibilities of administering these *β2m*^*ko*^ MKs in humans. With remarkable advances in techniques for generating clinical scale production of platelets from iPSCs^33^, platelets generated from iPSCs are likely to enter clinical studies in the near future. Further studies into identifying an optimal strategy for overcoming immune barriers will lead to broader applications of this technology.

## Materials and Methods

### Generation and characterization of iPSCs

Studies using human cells were approved by the Institutional Review Board of Faculty of Medicine, Chulalongkorn University (IRB# 212/58). All experiments were performed in accordance with relevant guidelines and regulations. Informed consent was obtained from the sample donors. Normal human iPSCs were generated by using episomal vectors. Briefly, 10^6^ peripheral blood mononuclear cells (PBMCs) were nucleofected with pCXLE-hOCT3/4-shp53-F (Addgene, 27077), pCXLE-hSK (Addgene, 27078), pCXWB-EBNA1 (Addgene, 37624) and pCXLE-hUL (Addgene, 27080). All plasmids were generous gifts from Shinya Yamanaka. Transfected PBMCs were seeded on mitotically inactivated MEF feeder cells in mTeSR1 feeder-free maintenance media for human ES and iPS cells (STEMCELL Technologies). After 21 days, iPSC colonies were picked and expanded for subsequent experiments.

iPSCs were then cultured in feeder-free conditions using Matrigel (Corning) and mTeSR1 feeder-free maintenance medium for human ES and iPS cells (STEMCELL Technologies), and they were maintained at 37 °C in a humidified atmosphere of 5% CO_2_. iPSCs were passaged using prepared CTK solutions (0.1 mg/ml collagenase IV, 0.25% (v/v) trypsin in PBS (-) supplemented with 20% (v/v) KSR, and 1 mM calcium chloride) for detaching cells. iPSCs were characterized by the detection of pluripotency marker expression using immunofluorescence (IF) staining and karyotype studies.

### Generation of tools for knocking out *β2m*

To knock out the *β2m* gene, paired CRISPR/Cas9 nickases and donor vectors were constructed for targeting and modifying exon 2. Single guide RNA sequences were designed by using the CRISPR Design Tool, which is available on the website http://crispr.mit.edu/ and was provided by the Zhang Lab (MIT, 2015). The guide sequences were cloned into a CRISPR plasmid backbone pSpCas9n (BB)-2A-Puro (PX462) from Feng Zhang (Addgene plasmid #48141) according to the target sequence cloning protocol of the Zhang Lab, and the targeting efficiency was detected with a T7 endonuclease 1 (T7E1) assay. The donor vector (Supplementary Fig. 1a), consisting of homologous arms (approximately 1,031 bp) and hygromycin-resistant gene components (approximately 2,161 bp), was constructed to disrupt β2m expression via homology-directed repair (HDR), as described in Supplementary Fig. 1b.

### Knocking out *β2m* in iPSCs

A total of 2.5 µg of each CRISPR/Cas9 nickase and 5 µg of the donor vector were transfected into a suspension of 5 × 10^6^ normal human iPSCs (O blood group) using 4D-Nucleofector X Unit-Transfection following the protocol for Human Stem Cells, P3 Primary cell 4D-Nucleofector X Kit (Lonza, V4XP-3012), using a CB-150 transfection program. As this transfection was performed under feeder conditions, MEF (DR4) ATCC SCRC-1045 cells were used as feeder cells, and mTeSR1 media with 20 µg/ml hygromycin was replaced at 72 hours post transfection. Genomic DNA was extracted from human iPSCs using a QIAamp DNA Blood Mini kit (Qiagen, Valencia, CA) according to the manufacturer’s instructions. PCR amplifications were carried out using Taq DNA polymerase (Fermentas, Glen Burnie, MD) and primers as described in Supplementary Table S1. PCR products were then sent for direct sequencing at Macrogen (Seoul, Korea) to confirm the recombination.

### Differentiation of iPSCs into HSCs, megakaryocytes and platelets

Human iPSCs were dissociated into small clumps of more than 100 cells per clump by treatment with CTK solution and then were transferred onto irradiated inactivated 10T1/2 cells. The cells were then cultured in haematopoietic cell differentiation medium: IMDM supplemented with a cocktail of 15% FBS, 2 mM L-glutamine, 5.5 µg/ml human transferrin, 10 µg/ml human insulin, 5 ng/ml sodium selenite, 0.45 mM α-monothioglycerol, 50 µg/ml ascorbic acid, and 20 ng/ml human vascular endothelial growth factor (VEGF). On day 14 of differentiation, cells were collected and gently disrupted with a pipette before being passed through 40-µm cell strainers. The obtained HSCs were transferred onto fresh irradiated or mitotically inactivated OP9 stromal cells, which were used as feeder cells; then, they were cultured in haematopoietic cell differentiation medium supplemented with 25 U/ml heparin, 100 ng/ml human thrombopoietin (hTPO; R&D systems), and 50 ng/ml human stem cell factor (hSCF; R&D systems) (Supplementary Fig. 1c). On days 21 and 24 of differentiation, loosely adherent cells and floating cells were collected by adding one-ninth volume of acid citrate dextrose solution, and the cells were centrifuged at 150 × g for 10 minutes to collect megakaryocytes for analysis. For analysis of platelet-like particles on day 24, one-ninth volume of acid citrate dextrose solution was added, and the cells were centrifuged at 150 × g for 10 minutes to precipitate any large cells, including megakaryocytes. Then, the supernatant containing the platelets was transferred to a new tube and centrifuged at 400 × g for 10 minutes to precipitate platelets and form pellets. GM6001, a broad-spectrum inhibitor of metalloproteases, was used to treat platelet cultures 2 days before analysis.

### Flow cytometry analysis

β2M and HLA class I cell surface expression on human iPSCs, HSCs, MKs and platelets was detected by staining with APC-conjugated anti-human β2-microglobulin (BioLegend) and PerCP-conjugated anti-human HLA-A, B, and C antibodies (BioLegend). For haematopoietic cell analysis on day 14 of differentiation, HSCs isolated from ES-sacs were stained with APC/Cy7-conjugated anti-human CD34 (BioLegend) and Brilliant Violet 421-conjugated anti-human CD45 antibodies (BioLegend). Megakaryocyte differentiation and platelet-like particles were detected by using FITC-conjugated anti-human CD41 (BioLegend) and PE-conjugated anti-human CD42b (BioLegend) staining. To assess platelet activation, platelets were stained with PE-conjugated anti-human CD62P antibody (P-selectin, BioLegend) and FITC-conjugated anti-human CD41 antibody (BioLegend) for 20 minutes at room temperature (RT). For polyploidy analysis of human iPSC-derived MKs, propidium iodide (PI) staining was performed. On day 21, iPSC-derived MKs were collected and fixed with 70% cold ethanol. Then, the cells were stained with PI staining solution (1 µg/ml PI in 1X PBS) containing 4 mM sodium citrate, 0.2 mg/ml RNase A and 0.1% Triton X-100. OP9 stromal cells were used as a 2N control group. The stained cells were analysed using a BD FACSAria II system (Becton Dickinson, Franklin Lakes, NJ).

### Immunofluorescence staining

For pluripotency marker detection, human iPSCs were fixed with 4% paraformaldehyde for 15–20 minutes at RT and then were permeabilized with 0.3% Triton X-100 diluted in 1X PBS for 15 minutes at RT. Cells were subsequently blocked with 10% goat serum for 30 minutes at RT and then were stained overnight at 4 °C with primary antibodies against Oct4 (Cell Signaling Technology), Nanog (Cell Signaling Technology), Ssea-4 (Abcam), Tra-1–60 (Cell Signaling Technology) and Tra-1–81 (Cell Signaling Technology). Cells were stained with an Alexa Fluor-conjugated secondary antibody (Life Technology, Invitrogen) and a FITC-conjugated secondary antibody (Millipore) for 1 hour at RT. DAPI was incubated with the cells for 15 minutes to stain the nuclei.

For embryoid body formation and detection of three germ layer markers, human iPSCs were dissociated, and several large clusters were used to form embryoid bodies in ultralow attachment culture dishes in an incubator that was at 37 °C with 5% CO_2._ On day 7, embryoid bodies were collected and placed on Matrigel-coated 24-well plates and then were cultured for 14 days at 37 °C in a 5% CO_2_ incubator. Then, the cells were fixed by incubation with 4% paraformaldehyde for 20 minutes at RT. Then, they were permeabilized by treatment with 0.3% Triton X-100 that was diluted in 1X PBS for 1 hour at RT. After that, cells were blocked by treatment with 10% goat serum for 1 hour at RT before being incubated at 4 °C overnight with primary antibodies for Brachyury (Abcam), alpha-fetoprotein (Abcam) and Nestin (BioLegend). Cells were stained with Alexa Fluor-conjugated secondary antibodies (Life Technology, Invitrogen) for 1 hour at RT. DAPI was used for nuclear staining for 15 minutes.

For proplatelet staining, iPSC-derived MKs on day 21 of differentiation were collected and reseeded onto coverslips coated with Matrigel and then were cultured for 24 hours. Subsequently, the cells were fixed with 4% paraformaldehyde for 15 minutes at RT and then were permeabilized with 0.3% Triton X-100 diluted in 1X PBS for 15 minutes at RT. After that, cells were blocked by incubation with 10% goat serum for 30 minutes at RT before being stained with anti-alpha-tubulin (Abcam) and Alexa Fluor 488 phalloidin (Molecular Probes, Invitrogen). Cells were mounted on glass slides with an antifade reagent containing DAPI (Invitrogen, Thermo Fisher Scientific), and imaging was performed on an EVOS FL colour Imaging System, Florescence Microscope (Life Technologies, Thermo Fisher Scientific).

### Wright’s staining

iPSC-derived MKs on day 21 of differentiation were treated with Wright stain solution and were analysed under a microscope (Olympus, CH20i) to detect the morphology of cells, including their size and presence of multilobed nuclei.

### Platelet activation and aggregation assays

Human blood-derived platelets and iPSC-derived platelets were prepared according to the protocol from Takayama *et al*., 2008^34^. Briefly, iPSC-derived platelets in culture medium were gently collected as described above. The supernatant was transferred to a new tube with 1 µM PGE1 to prevent platelet activation, and then it was centrifuged at 400 × g for 10 minutes to obtain platelet pellets. The pellets were washed with modified Tyrode-HEPES buffer at pH 7.4 (10 mM HEPES, 12 mM NaHCO_3_, 138 mM NaCl, 5.5 mM glucose, 2.9 mM KCl, and 1 mM MgCl_2_, pH 7.4) in an appropriate volume, and then they were centrifuged at 400 × g for 10 minutes. The washed platelet pellet was resuspended in modified Tyrode-HEPES buffer at pH 7.4 with 1 mM CaCl_2_, and then it was incubated at 37 °C for 0.5–1 hours before functional tests were performed.

Platelets were stimulated with 50 µM ADP (Adenosine diphosphate, R&D system) and 1 U/ml thrombin (Sigma), and then they were incubated for 1 minute at 37 °C. Then, the cells were fixed immediately with 1% paraformaldehyde at 4 °C for at least 30 minutes before being washed with cell staining buffer (3% FBS in PBS). Platelet activation was assessed by flow cytometry after cell staining.

Washed FITC-conjugated anti-human CD41 (BioLegend)-labelled human blood-derived platelets were mixed with washed PE-conjugated anti-human CD42b (BioLegend)-labelled blood-derived platelets or iPSC-derived platelets in a 1:1 proportion, and then they were stimulated with 50 µM ADP (Adenosine diphosphate, R&D system) and 1 U/ml thrombin (Sigma). These platelets were incubated for 20 minutes at 37 °C to trigger platelet aggregation. Then, flow cytometry was used to assess dual-coloured events that represented platelet aggregation. Nonstimulated platelets were used as a control group.

### *In vivo* platelet survival assay

NSG mice were acquired from the Jackson Lab (Maine, USA). The animal protocol was approved by the animal IRB of Chulalongkorn University (#1573009). All methods were performed in accordance with the relevant guidelines and regulations. Eight-week-old NSG mice were injected with clodronate liposomes 24 hours before MK injections. One million MKs (wild-type or *β2m*^*ko*^) were intravenously administered to the mice, and there were four mice in each group. Two and 24 hours after the administration of the MKs, PBMCs were collected from the mice. The percentages of human platelets present were determined using human CD41 and human CD42b as indicators that were measured by flow cytometry.

## Supplementary information

Supplementary Information.

## Data Availability

The data are available upon request.
