# Supplementary material for: Generation and characterization of HLA-universal platelets derived from induced pluripotent stem cells
Source: Sci Rep. 2020 May 21;10:8472. doi: 10.1038/s41598-020-65577-x (PMC7242456; doi:10.1038/s41598-020-65577-x)
Supplement: Supplementary file 1 — Supplementary Information. [file 41598_2020_65577_MOESM1_ESM.pdf]

Supplementary Information

Generation and characterization of HLA-universal platelets derived from induced pluripotent stem cells

Phatchara Norbnop[1,2], Praewphan Ingrungruanglert[3,4], Nipan Israsena[3,4], Kanya Suphapeetiporn[2,5]*, Vorasuk Shotelersuk[2,5]

[1]Doctor of Philosophy Program in Medical Sciences, Faculty of Medicine, Chulalongkorn University, Bangkok 10330, Thailand

[2]Center of Excellence for Medical Genomics, Department of Pediatrics, Faculty of Medicine, Chulalongkorn University, Bangkok 10330, Thailand

[3]Stem Cell and Cell Therapy Research Unit, Faculty of Medicine, Chulalongkorn University, Bangkok 10330, Thailand

[4]Department of Pharmacology, Faculty of Medicine, Chulalongkorn University, Bangkok 10330, Thailand

[5]Excellence Center for Genomics and Precision Medicine, King Chulalongkorn Memorial Hospital, the Thai Red Cross Society, Bangkok, 10330, Thailand

Supplementary Table S1

Primers used for detecting left and right donor recombination and wild-type alleles.

Alleles	Forward primers	Reverse primers
**Left donor recombination**	5’-TGGAAGGGGTGGAAACAGAG-3’	5’-ATGTGGAATGTGTGCGAGGC-3’
**Right donor recombination**	5’-AAGACAATAGCAGGCATGCTG-3’	5’-TGGTTAGAAATAAGGCTGGC-3’
**Wild type**	5’-GGGAGAAATCGATGACCAAA-3’	5’-CCCTGACAATCCCAATATGC-3’

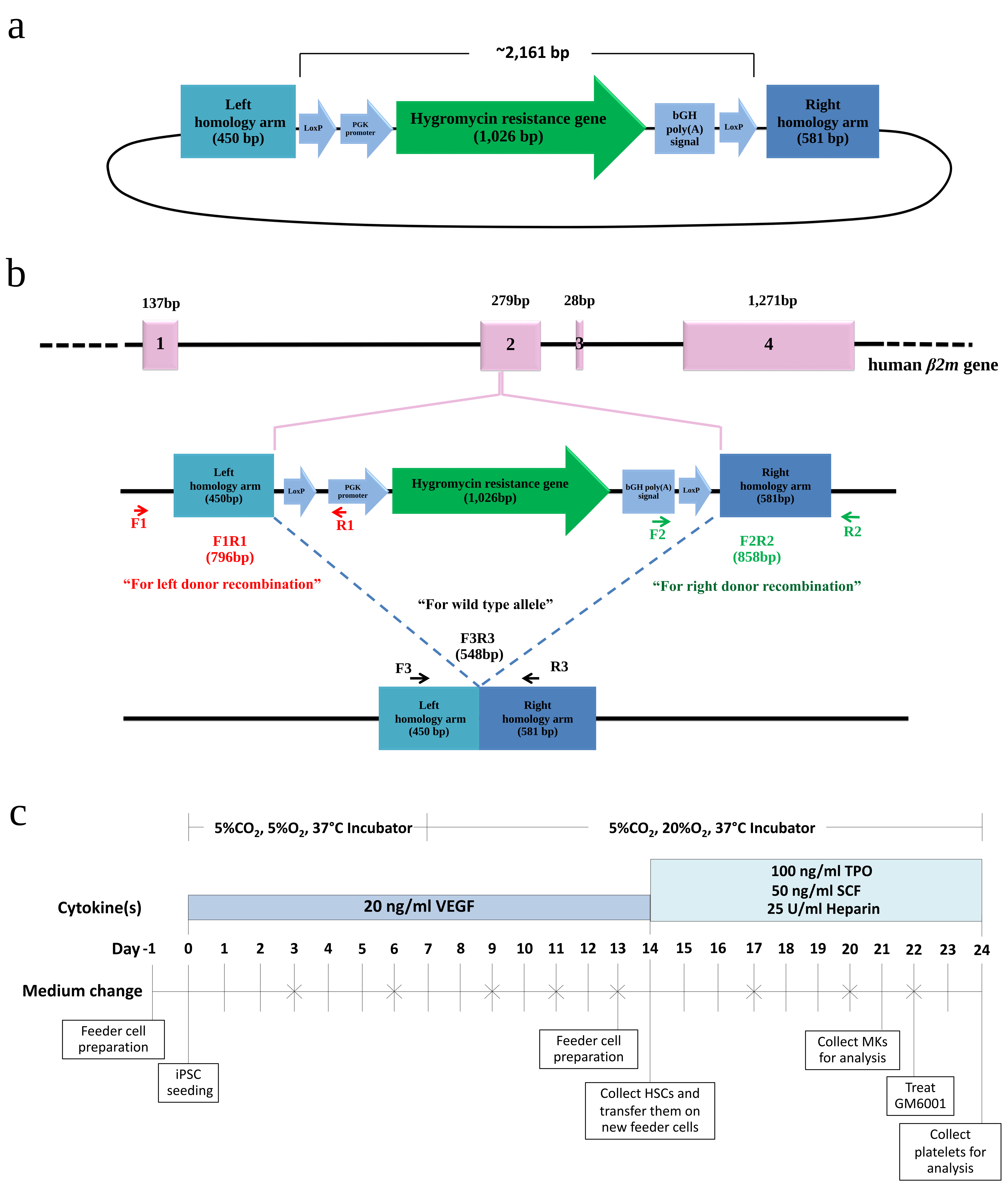

Supplementary Figure 1. Schematic diagram illustrates the donor vector used to knock out the β2-microglobulin (*β2M*) gene (**a**). Abbreviations: left homology (HL) arm, right homology (HR) arm, PGK promoter (PGK), hygromycin resistance gene (HygR), bGH poly (A) signal (bGH polyA). The *β2M* allele with donor recombination and without donor recombination and primer pairs used for detection (**b**). Schematic diagram represents an *in vitro* megakaryocyte (MK) and platelet differentiation protocol from sac-like structures (ES-sac method) (**c**).

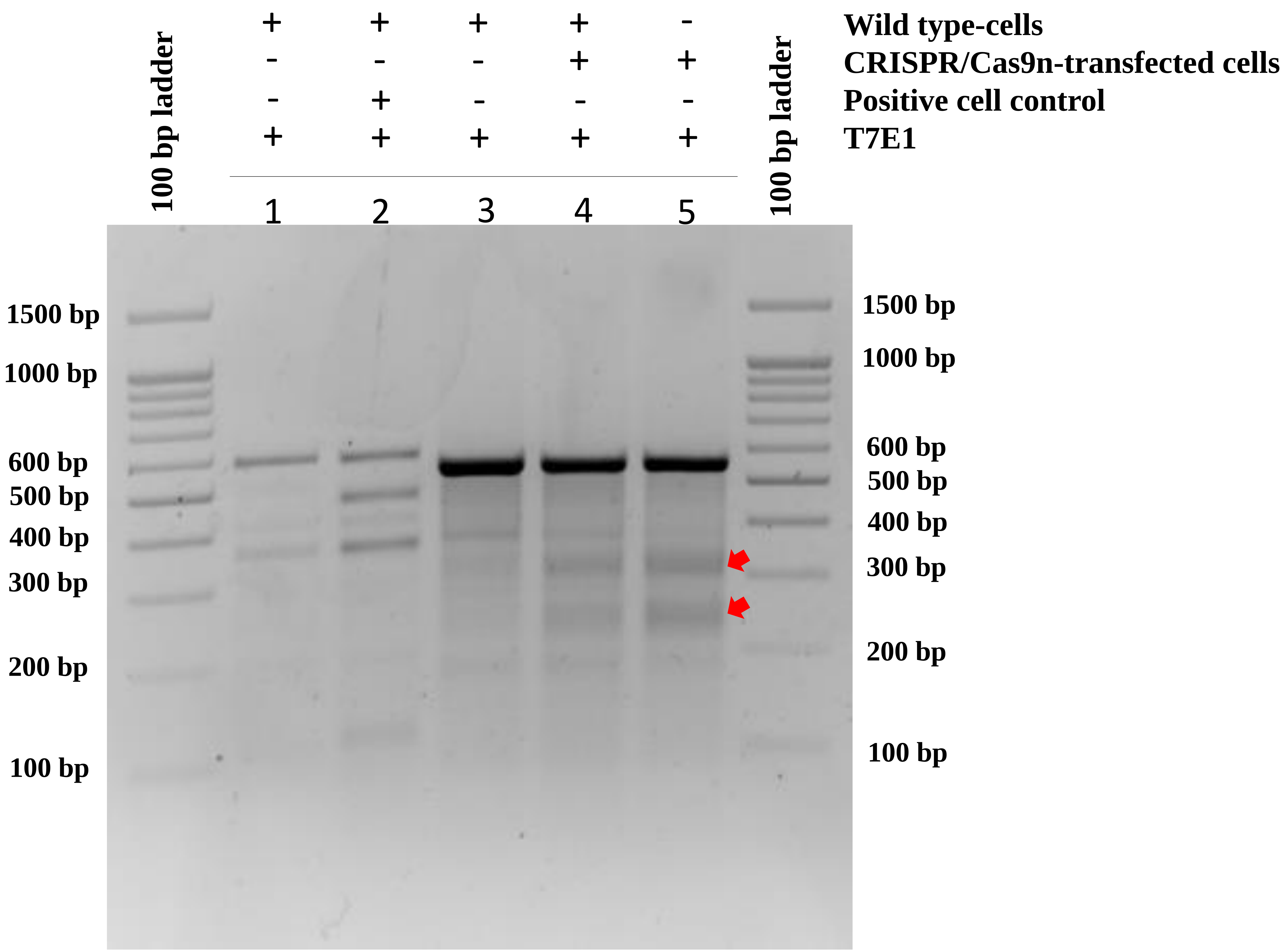

Supplementary Figure 2. Gel electrophoresis showing the product of CRISPR/Cas9 nickase detected by T7E1 assay. A 100-bp ladder was used for size determination.

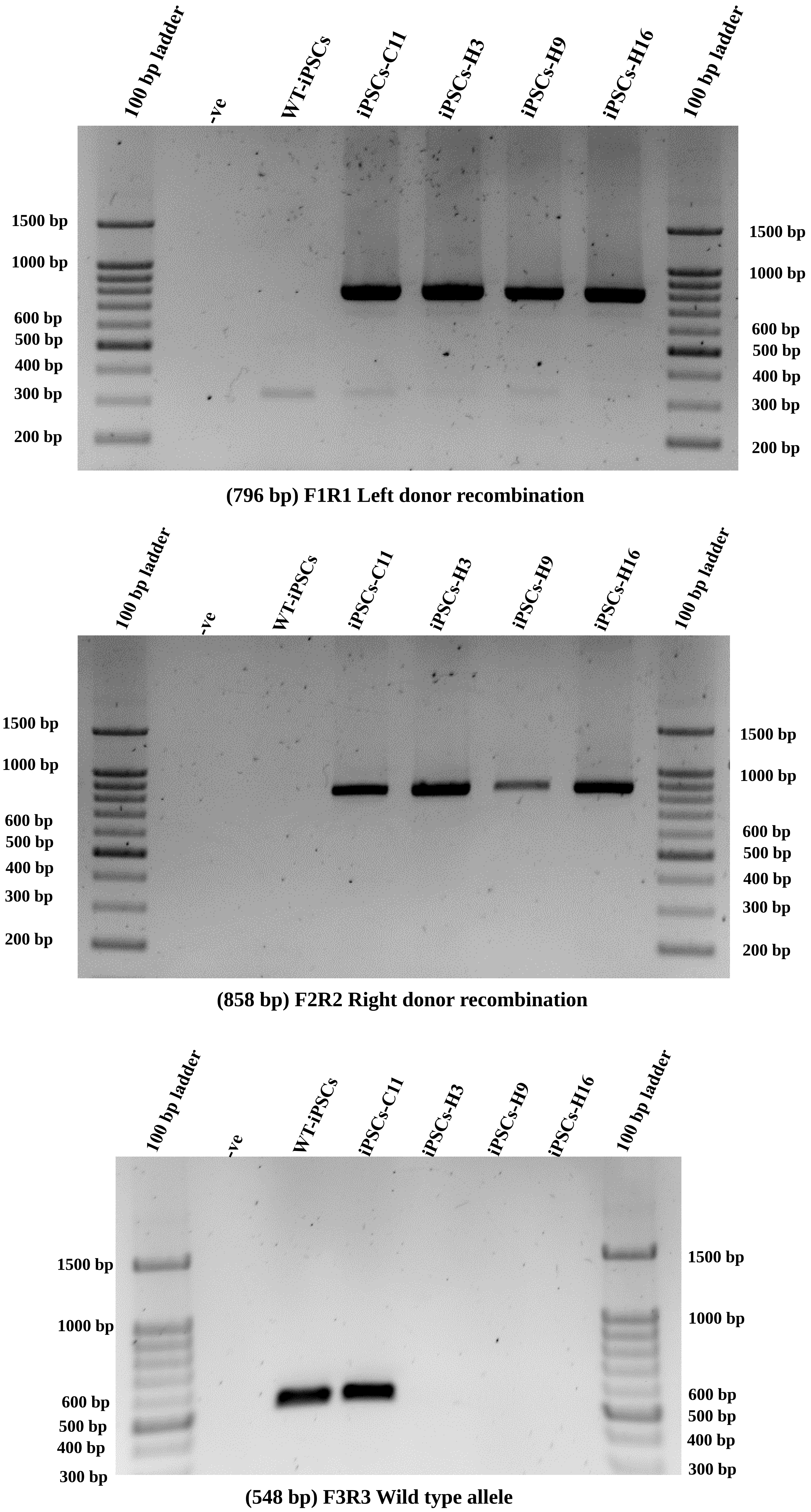

Supplementary Figure 3. PCR amplification and gel electrophoresis results of donor recombination in all iPSC lines for iPSC characterization. A 100-bp ladder was used for size determination.

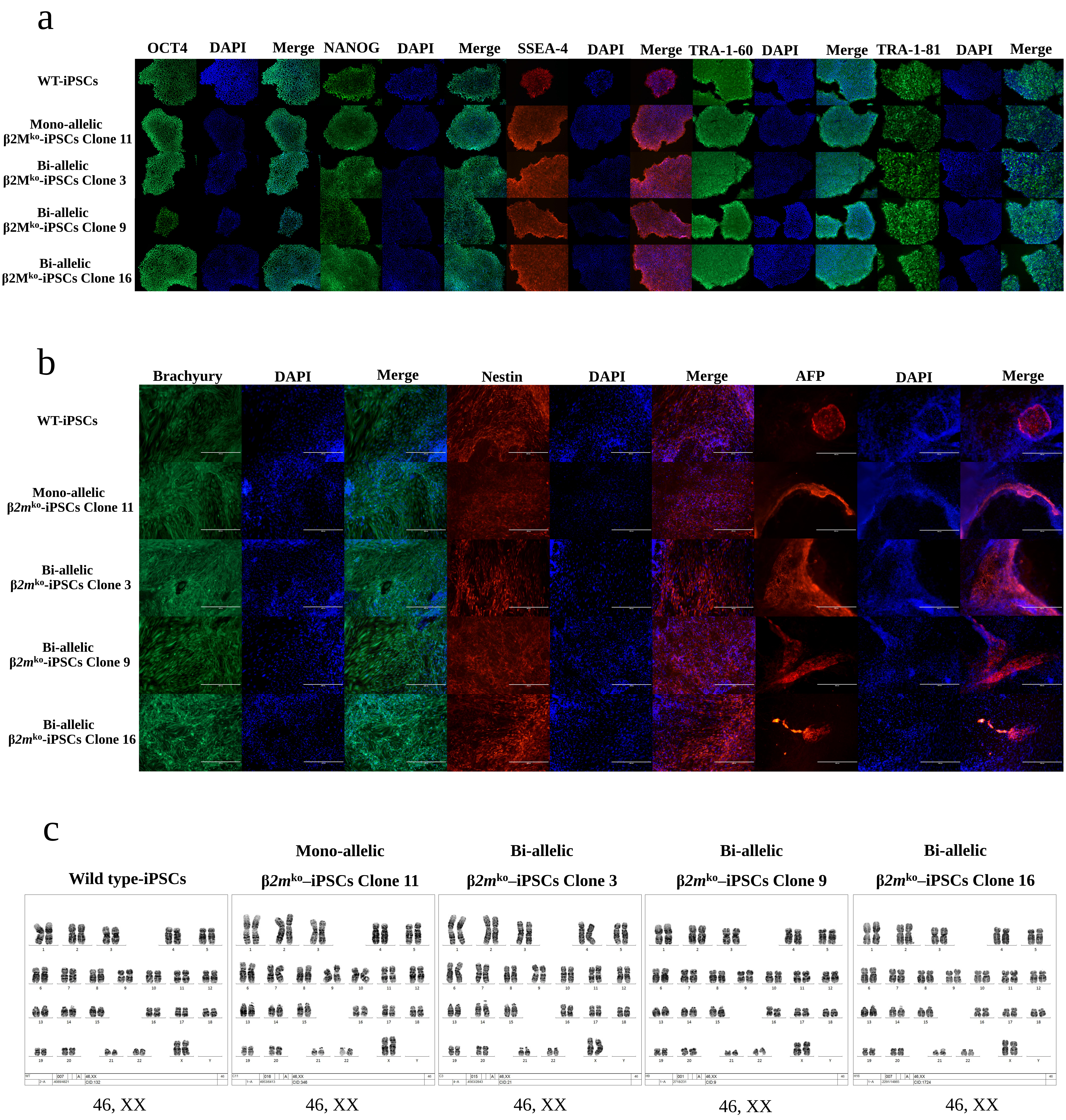

Supplementary Figure 4. Immunofluorescence staining showing expression of pluripotency markers: OCT4, NANOG, SSEA-4, TRA-1-60, and TRA-1-81 (**a**); staining for α-fetoprotein (endoderm), Brachyury (mesoderm) and Nestin (ectoderm). DAPI (blue) was used for nuclear staining (**b**). All iPSC lines were pluripotent. **(c)** All iPSC lines had normal karyotypes.

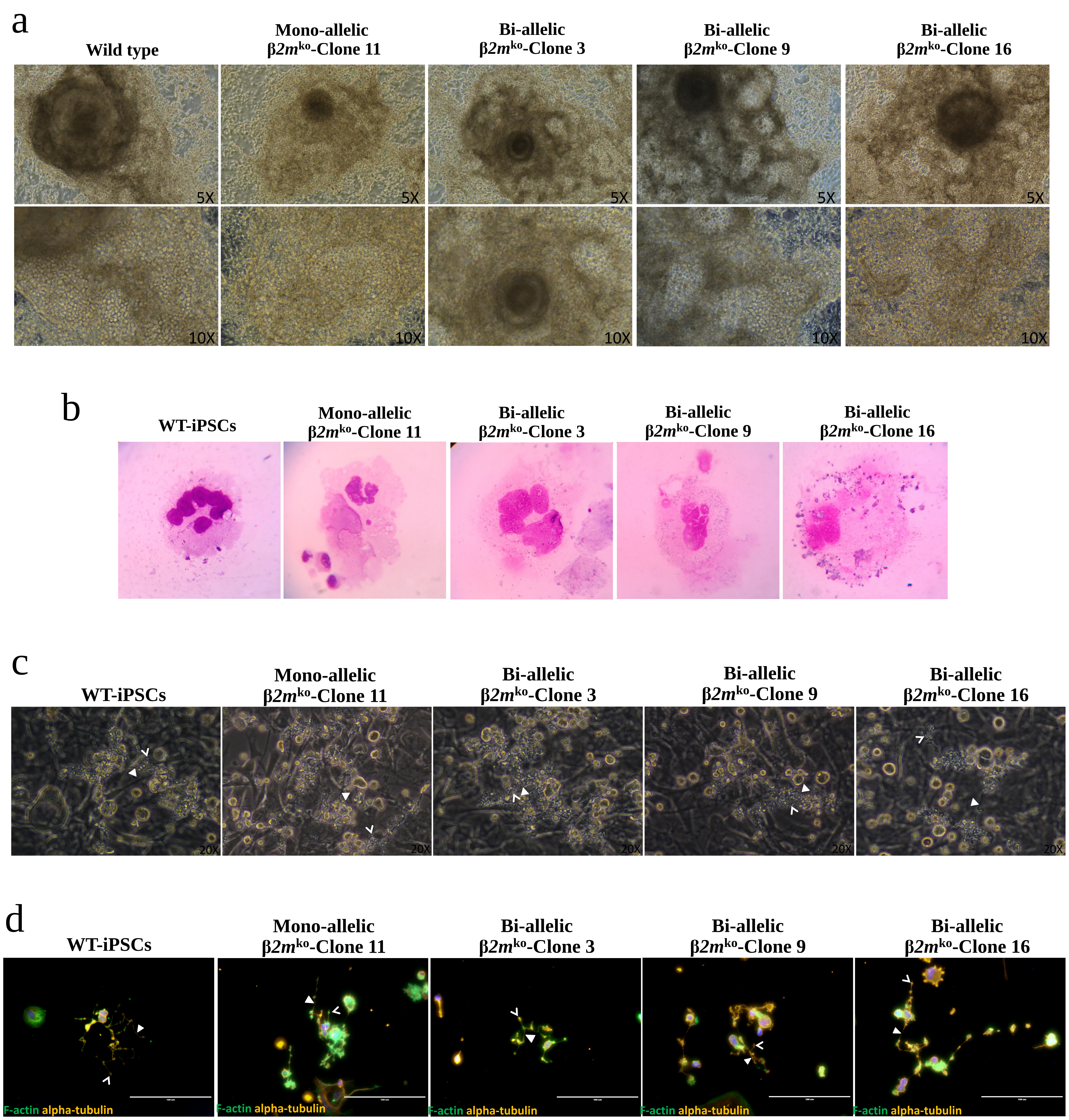

Supplementary Figure 5. Characterization of haematopoietic stem cells by flow cytometry on day 14. Phase contrast photomicrographs of ES-sacs generated from wild-type iPSCs, monoallelic *β2M*^{ko}-iPSCs and three lines of biallelic *β2M*^{ko}-iPSCs; magnification 5X and 10X (**a**). MK morphology using Wright's staining (**b**). Megakaryocyte and platelet characterization by proplatelet formation. Phase contrast photomicrographs show megakaryocyte colonies with proplatelet formation on day 21 of culture (**c**) and immunofluorescence staining in the proplatelet formation assay (**d**).
